# Study on the Effect of the Three-Dimensional Electrode in Degradation of Methylene Blue by Lithium Modified Rectorite

**DOI:** 10.1155/2016/8198235

**Published:** 2016-11-16

**Authors:** Jian Huang, Yin'an Ming, Ying Du, Yingru Wang, Ci'en Wang

**Affiliations:** School of Chemistry Environmental Engineering, Wuhan Institute of Technology, Wuhan 430073, China

## Abstract

This study presents the electrochemical degradation of methylene blue (MB) wastewater in a synthetic solution using three-dimensional particle electrodes. The novel particle electrodes were fabricated in this work using the lithium modified rectorite (Li-REC). The adsorption property of the fabricated particle electrodes was studied in a series of experiments. The optimum electrochemical operating conditions of plate distance, cell voltage, and concentration of electrolyte were 2 cm, 9 V, and 0.06 mol L^−1^, respectively. It was also found that microwave irradiation can effectively improve the adsorption property and electrical property of the fabricated electrodes. In addition, the scanning electron microscope (SEM) of the fabricated electrodes was investigated. The experimental results revealed the order of adsorption property and electrical property of the fabricated electrodes. So, fabricated electrodes are not only of low cost and mass produced, but also efficient to achieve decolorization of MB solution.

## 1. Introduction

In recent years, with the development of society, the problem of environment pollution becomes more and more serious. One of the most environmental concerns is dyes in wastewater from industries producing paper, leather, textiles, and so forth. However, several of dyes and their derivatives not only are toxic and carcinogenic to mankind, cause allergy and dermatitis [1, 2], and even provoke cancer [3], but also are nonbiodegradable; methylene blue (MB) is a sort of typical dyes [4]. Therefore, how to remove such dye from effluents for avoiding adverse health hazards and protecting the ecosystem is a matter of great urgency.

There are many researches which have drawn great attention to degradation of dyes in wastewater. It can be achieved by using chemical [5, 6], physical [7, 8], and biological [9, 10] treatment techniques. Some methods with both physical and biological treatment are usually environmental friendly [11]; they are associated with certain flaws [12] and the low removal efficiency, especially some industrial wastewater containing nonbiodegradable dyes [13]. It is known that the chemical treatment techniques can be more efficient, but complete degradation is normally costly and sometimes it may bring new pollutants in system [14].

Thus, these limitations have spurred an effort to develop effective and environmentally friendly treatment of dye effluents. A new electrochemical reactor, three-dimensional electrode reactor, has drawn much attention by its large specific surface area and high efficiency when compared with conventional two-dimensional electrodes [15, 16]. Also, the conventional particle electrodes such as activated carbon [17] and ceramic particles [18] are both costly and difficult to regenerate. So, the paper describes a synthetic approach of the novel particle electrodes made by lithium modified rectorite (Li-REC), which are of low cost, mass produced, and found to be efficient for such application.

Rectorite (REC) is a regularly interstratified clay mineral composed of alternating pairs of a nonexpansible dioctahedral mica-like layer, and an expansible dioctahedral smectite-like layer in a 1 : 1 ratio [19]. The interlayer cations, Na^+^, K^+^, and Ca^2+^ and so forth in the smectite-like layers, can be easily exchanged with either organic or inorganic cations [20–24]. Therefore, with the smaller ionic semidiameter and good electrical conductivity of lithium, the lithium modified rectorite is characterized by good adsorption and electrical property. Furthermore, with the function of polyvinyl alcohol (PVA), the fabricated particle electrodes not only have good adsorption and electrical property but also have high mechanical strength.

The novel particle electrodes were prepared and applied to the degradation of a synthetic solution containing MB as model in three-dimensional electrode reactor. Compared with conventional particle electrodes, these electrodes are of low cost, mass produced, and found to be efficient for such application. And the fabricated electrodes were characterized by scanning electron microscope (SEM). Furthermore, the effects of two parameters (microwave power and microwave irradiation time) on the fabricated particles adsorption property were discussed. The study shows that these fabricated particle electrodes not only have good electrochemical and adsorption properties but also can be efficient on MB removal.

## 2. Experiment

### 2.1. Materials

Rectorite was supplied by the Rectorite Deposit of Zhongxiang, Hubei, China. Lithium carbonate (Li_2_CO_3_) was purchased from Shanghai Zhanyun Chemical Reagent Factory. Oxalic acid (C_2_H_2_O_4_·H_2_O), boric acid (H_3_BO_3_), and anhydrous calcium chloride (CaCl_2_) were provided by Tianjing Bodi Chemical Reagent Factory. Methylene blue (MB), anhydrous sodium sulfate (Na_2_SO_4_), polyvinyl alcohol (PVA), sodium alginate (SA), and hexane diamine (NH_2_(CH_2_)_6_NH_2_) were purchased from Sinopharm Chemical Reagent Factory. All chemicals were used as received without further purification. Distilled water was used in the experiments.

### 2.2. Particle Electrodes Preparation

4 g PVA was thoroughly dissolved in 30 mL distilled water in the process of heating in water bath at 95°C for 40 mins in a beaker; then it is cooled down to room temperature (25°C). After that, 0.06 g sodium alginate (SA) and 6 g lithium modified rectroite (Li-REC) were added to the solution with sufficient agitatation until being well blended. The mixture was molded into many small spherical particles (3-4 mm diameter) and dropped into the 2% calcium chloride (CaCl_2_) saturated boric acid (H_3_BO_3_) solution by 10 mL disposable syringe, staying inside for 4 h. Then, the particles were pulled out, filtered, and washed with distilled water and put into the 5% (v/v) hexane diamine (NH_2_(CH_2_)_6_NH_2_) solution for 1 h solidifying. Finally, the particles were washed with distilled water at least three times and then oven-dried for 8 h at 65°C. Another particle electrodes made by raw rectroite (REC) were also prepared with the same procedures as a blank control. The particle electrodes made by Li-REC and raw REC were marked as S_1_ and S_2_, respectively.

In order to obtain larger specific surface area and improve the adsorption abilities of these particles, further microwave modification experiments were done according to the two factors (microwave power and microwave irradiation time). Therefore, two new samples were obtained marked as S_3_ and S_4_, respectively, by microwave irradiating the sample S_1_ and S_2_.

The surface of the samples S_1_ and S_2_ were examined by scanning electron microscope (SEM) (Hitachi, S-750, Japan). In addition, a series of experiments of adsorption and electrochemistry on MB removal were made to show the adsorption and electrical properties of the fabricated particles.

### 2.3. Experimental Setup

**Figure 1:**
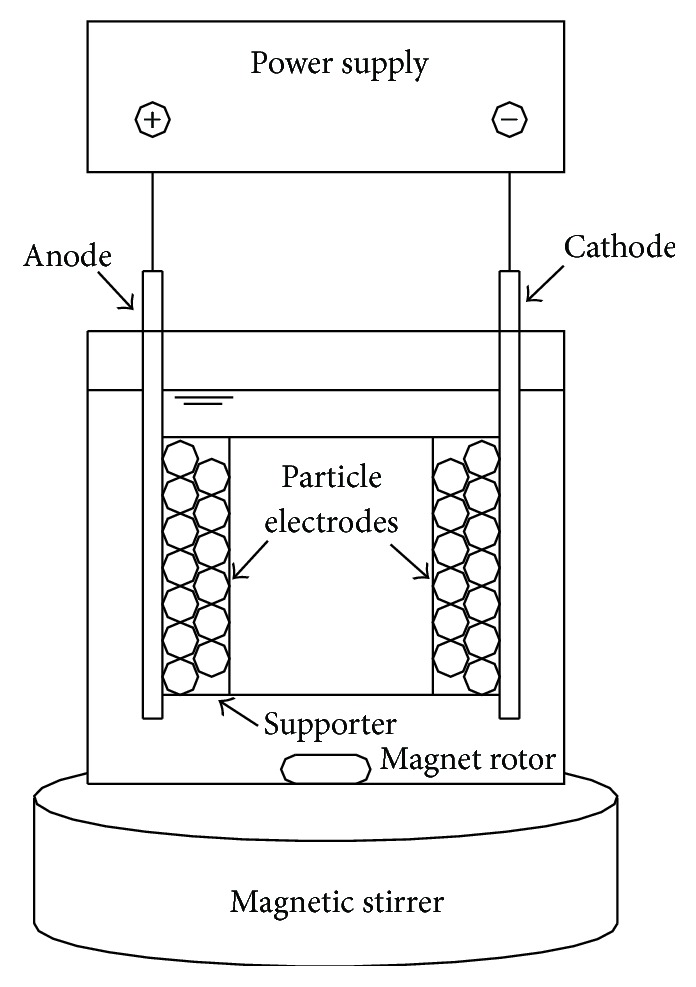
Schematic diagram of experimental setup.

The three-dimensional reactor was designed with a working volume of 250 cm^3^ (Figure 1). Titanium-ruthenium (90 mm *∗* 50 mm *∗* 2 mm) and stainless steel (90 mm *∗* 50 mm *∗* 2 mm) were used as anode and cathode, respectively. The simulative wastewater was prepared by dissolving 0.4 g·L^−1^ of MB in distilled water with 8.52 g·L^−1^ Na_2_SO_4_ as electrolyte. The electric power was supplied with regulated DC power supply (WSA-H, Shenzhen, China). Magnetic stirrer and magnet rotor were used to ensure better mixing effects. In order to eliminate the influence of the adsorption of target compound, the particle electrodes were firstly soaked in MB simulated wastewater until adsorption saturation and then filled between the main electrodes.

### 2.4. Bath Experiments

#### 2.4.1. Adsorption Experiment

Adsorption experiments were conducted using 250 mL glass beaker containing the 200 mL MB dye (400 mg·L^−1^), 1.704 g Na_2_SO_4 _(0.06 mol·L^−1^), and 1 g fabricated particles. The glass beaker containing the mixture solution was placed on a magnetic stirrer and with the stirring of magnet rotor at room temperature for 3 h. At every 30 mins intervals during the reaction, 1 mL of the reaction solution was quickly sampled into 100 mL volumetric flask by 1 mL pipette and then was diluted 100 times by distilled water. The diluent was analyzed to determine MB concentrations of the solution by UV/Vis spectrometry at 665 nm. The amount of MB adsorbed onto the fabricated particles was determined by the difference between the initial and remaining concentration of MB solution. The adsorption capacity (*Q*) was calculated as

(1) $$\begin{array}{c} Q=\left(\;C_{0}-C_{t}\;\right)\frac{V}{W}, \end{array}$$

where *C*_0_ and *C*_*t*_ refer to the initial and time *t* concentrations of MB solution (mg·L^−1^), while *W* and *V* are the quality (g) of fabricated particles and the volume of MB solution (L), respectively.

#### 2.4.2. Electrochemical Experiments

Figure 1 shows the schematic of three-dimensional electrodes reactor, and without adding the fabricated particle electrodes, it was conventional two-dimensional electrodes. In order to ensure the optimal operating conditions of MB removal for electrochemical experiments, the effects of various parameters (plate distance, cell voltage, and concentration of electrolyte) on the MB removal efficiency were investigated. Through a series of experiments the optimal experimental conditions with plate distance, cell voltage, and concentration of electrolyte were identified as 2 cm, 9 V, and 0.06 mol·L^−1^, respectively.

With the optimal experimental conditions mentioned above, 1 g fabricated particle electrodes and another 1 g fabricated electrodes which exposed to enough MB solution were filled between the main electrodes, respectively. With the DC power supply, every fabricated particle was polarized and behaved as an anode on one side and a cathode on the other side. At every 30 mins intervals during the 3 h electrochemical experiment, 1 mL of the reaction solution was quickly sampled into 100 mL volumetric flask by 1 mL pipette and then was diluted 100 times by distilled water. The diluent in volumetric flask was analyzed to determine MB concentrations of the solution by UV/Vis spectrometry at 665 nm. The removal rate was calculated by following equation:

(2) $$\begin{array}{c} \text{Removal rate}=\frac{\left(C_{0}-C_{t}\right)}{C_{0}}\times 100\%, \end{array}$$

where *C*_0_ and *C*_*t*_ are the concentrations of MB (mg·L^−1^) before electrochemical experiment and after an experiment time *t*, respectively.

## 3. Results and Discussion

### 3.1. Characterization of Particle Electrodes

**Figure 2:**
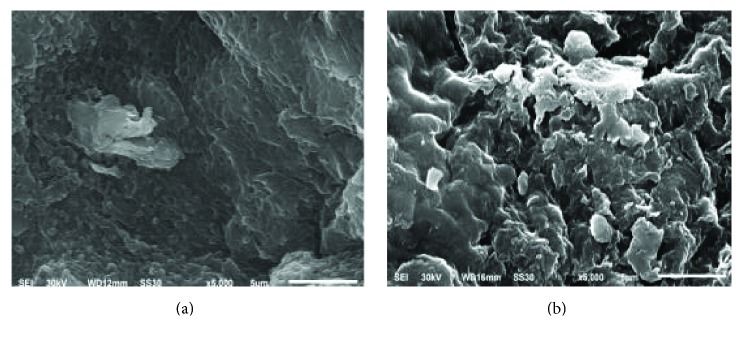
The SEM of rectorite particles.

SEM patterns of sample S_1_ and S_2_ were shown in Figure 2. Figures 2(a) and 2(b) show the SEM magnified 5000 times of the fabricated particles S_1_ and S_2_, respectively. More porosity can be seen in sample S_1_ than in sample S_2_, and the porous structure might greatly strengthen the mass transfer, which can accelerate the reaction rate. It was also found that the fabricated particles do not change the layered structure of rectorite. The stability in mechanical properties of the fabricated particle electrodes was confirmed.

### 3.2. Effect of Microwave Power on Adsorption

The adsorption was influenced by the power of microwave. It can be seen from Table 1 that the adsorptive reactions were directly proportional to the power of microwave. The effect of the microwave power on MB adsorption was carried out by adding 400 mg·L^−1^ concentration of MB solution (200 mL) and 1 g fabricated particles (S_4_) with the stirring of magnet rotor at room temperature for 3 h. And all the microwave irradiation time of the adsorbents is the same with different microwave power. It was also found in Table 1 that the particle electrodes adsorption capacity increased from 13.91 mg·g^−1^to 23.62 mg·g^−1^which almost doubled, and the removal rate increased from 22.65% to 26.58% with the increase of microwave power from 300 w to 800 w, respectively. Through the different irradiation temperature caused by different power, the particles may become more porous with the higher temperature, so both of the adsorption capacity and removal rate were increased. However, higher power leads to more power consumption. When taking into account that the differences of removal rate between 500 w and 800 w are not significant, the power of 500 w was used for irradiation.

### 3.3. Effect of Irradiation Time on Adsorption

The effect of the microwave irradiation time on MB adsorption was carried out by adding 400 mg·L^−1^ concentration of MB solution (200 mL) and 1 g fabricated particles (S_4_) with the stirring of magnet rotor at room temperature for 3 h. The results are described in Table 2, between 5 and 30 mins; the minor differences of adsorption capacity and removal rate fluctuate across 22.43 mg·g^−1^ and 25.07%, respectively. The adsorption capacity and removal rate of 30 mins sharply decreased to 19.02 mg·g^−1^ and 21.63%, respectively. Although the adsorbent had a stable layered structure and the porosity of the fabricated particles would be increased under irradiation of microwave, the layered structure was damaged at high temperature caused by long time irradiation. Meanwhile, in consideration of power consumption, the irradiation time was identified as 10 mins.

### 3.4. Adsorption Study

**Figure 3:**
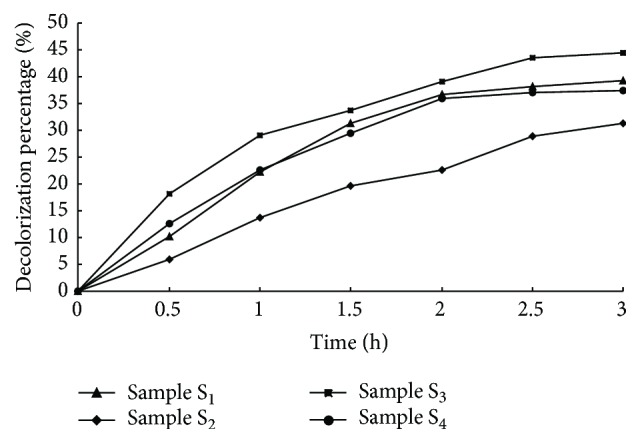
The adsorption property of fabricated particles.

As mentioned above, samples S_3_ and S_4_ were obtained by microwave irradiating (500 w, 5 min) samples S_1_ and S_2_, respectively. With the stirring of magnet rotor, 1 g of adsorbents (S_1_, S_2_, S_3_, and S_4_) was added to 200 mL MB solution with known concentration of 400 mg·L^−1^ at room temperature, respectively. The optimum time for the adsorption process was selected as 3 h, and every 30 mins intervals 1 mL reaction solution was sampled for testing absorbance by UV/Vis spectrophotometer. The results were described in Figure 3. It was found that the order of removal rate of particles on MB removal is S_3_ > S_1_ > S_4_ > S_2_. As known from above, samples S_1_ and S_2_ were made by lithium modified rectorite (Li-REC) and raw rectorite, respectively. The sample S_1_ had a rough surface with porous structure than sample S_2_ which could be drawn from Figure 2. So with the larger layered space of Li-REC, the adsorption property of sample S_1_ is better than S_2_. Meanwhile, the particles become more porous after microwave irradiation, so the adsorption property of samples S_3_ and S_4_ is better than S_1_ and S_2_, respectively.

### 3.5. Electrochemical Degradation Studies

**Figure 4:**
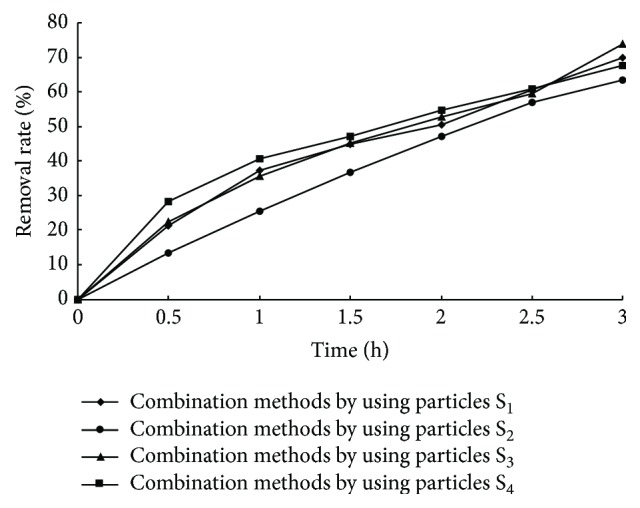
Degradation of MB solution with combination methods.

**Figure 5:**
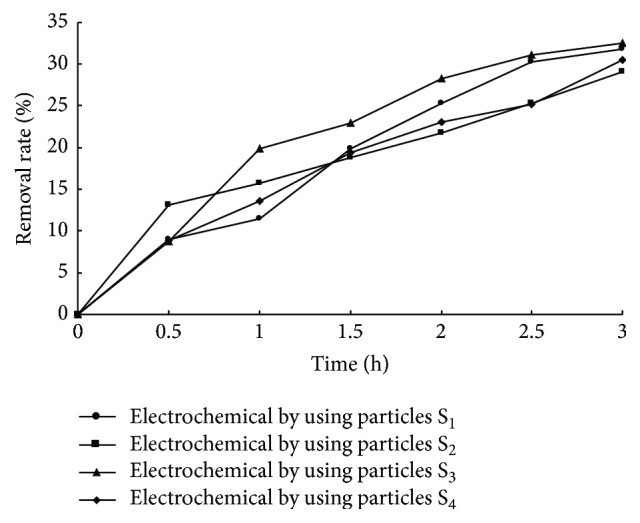
Degradation of MB solution with three-dimensional electrode.

A series of comparable experiments were performed for optimal experimental conditions with plate distance, cell voltage, and concentration of electrolyte of 2 cm, 9 V, and 0.06 mol·L^−1^, respectively. And the MB removal rate of two-dimensional electrode reactor is 27.1%. To compare with two-dimensional and three-dimensional electrochemical degradation on the same optimal electrochemical experimental conditions mentioned above, 1 g adsorbents and 200 mL MB solution (initial concentration 400 mg·L^−1^) were added to the electrolytic devices separately, and the tests were carried out at room temperature for 3 h. Total removal rates of MB solution with the combination methods of adsorption and electrochemical were shown in Figure 4, and degradation of three-dimensional electrochemical with the adsorption saturation fabricated particles was shown in Figure 5.

It can be clearly seen from Figure 4 that all the removal rates of MB solution by using the combination methods with different fabricated electrodes were increased with reaction time. The total removal rates were 70.0%, 63.32%, 73.7%, and 67.68% by using the fabricated particle electrodes S_1_, S_2_, S_3_, and S_4_, respectively. As known from the adsorption study mentioned above, the removal rates on MB removal of the adsorption of S_1_, S_2_, S_3_, and S_4_ are 39.34%, 31.38%, 44.45%, and 37.48%, respectively. And the removal rate of two-dimensional electrode electrochemistry was 27.1%. So the MB removal rates of the combination methods were greater than the sum of the removal rate of particles adsorption and electrochemical methods. Thus, the fabricated particles could act as three-dimensional particulate electrodes. In order to confirm the guessing and eliminate the influence of the adsorption of MB, the particle electrodes were firstly soaked in MB simulated wastewater until adsorption saturation and then filled between the main electrodes. It can be seen from Figure 5 that the order of removal rate on MB removal is S_3_ (32.49%) > S_1_ (31.78%) > S_4_ (30.48%) > S_2_ (29.03%). Meanwhile, the MB removal rates were still greater than the removal rate of two-dimensional electrode (27.1%). So it could be confirmed that the fabricated particles not only had better adsorption property but also can act as three-dimensional particulate electrodes. Moreover, the cost of these electrodes was lower. As consideration on mass production, it was found to be efficient for such application.

## 4. Conclusion

This study presents the removal of MB in simulated wastewater by three-dimensional electrode reactor with fabricated particles made mainly by Li-REC and serviced as particle electrodes. The fabricated particle electrodes are beneficial to increase contact area of the electrochemical reactor. The effect of microwave power and microwave irradiation time on the fabricated particle electrodes for MB removal efficiency was investigated, and the optimum power and irradiation time are optioned to be 500 w and 10 mins, respectively. It is demonstrated that the order of adsorption property and electrical property of the fabricated particles is S_3_ > S_1_ > S_4_ > S_2_. And it can be confirmed that the fabricated Li-REC particles modified by microwave not only have good adsorption property but also can act as three-dimensional particulate electrodes. Moreover, these electrodes are of low cost, mass produced, and found to be more efficient for such application. Therefore, the fabricated Li-REC particles modified by microwave can play an important role in the three-dimensional electrodes system. Furthermore it can be regarded as a viable alternative for the treatment of MB wastewater.

## Figures and Tables

**Table 1:** Adsorption capacity and removal rate of particle electrodes on MB removal with the effect of microwave power.

Microwave power (w)	Adsorption capacity (mg·g^−1^)	Removal rate (%)
300	13.91	22.65
500	20.28	25.65
800	23.62	26.58

**Table 2:** Adsorption capacity and removal rate of particle electrodes on MB removal with the effect of microwave irradiation time.

Microwave irradiation time (min)	Adsorption capacity (mg·g^−1^)	Removal rate (%)
5	22.22	24.87
10	22.43	25.07
15	22.76	25.03
30	22.33	25.15
45	19.02	21.63
